# Cardiovascular disease management during the coronavirus disease 2019 pandemic

**DOI:** 10.7150/ijms.46484

**Published:** 2020-05-29

**Authors:** Wen-Hsien Lee, Ying-Chih Chen, Szu-Chia Chen, Chang-Jen Chen, Po-Chao Hsu, Wei-Chung Tsai, Chun-Yuan Chu, Chee-Siong Lee, Tsung-Hsien Lin, Wen-Chol Voon, Chao-Hung Kuo, Ho-Ming Su

**Affiliations:** 1 Department of Internal Medicine, Kaohsiung Municipal Siaogang Hospital, Kaohsiung, Taiwan.; 2 Faculty of Medicine, College of Medicine, Kaohsiung Medical University, Kaohsiung, Taiwan.; 3 Division of Cardiology, Department of Internal Medicine, Kaohsiung Medical University Hospital, Kaohsiung, Taiwan.; 4 Regenerative Medicine and Cell Therapy Research Center, Kaohsiung Medical University, Kaohsiung, Taiwan.

**Keywords:** coronavirus, cardiovascular, pandemic

## Abstract

Based on clinical presentation, pathophysiology, high infectivity, high cardiovascular involvement, and therapeutic agents with cardiovascular toxicity of coronavirus disease 2019 (COVID-19), regular cardiovascular treatment is being changing greatly. Despite angiotensin-converting enzyme 2 serving as the portal for infection, the continuation of clinically indicated renin-angiotensin-aldosterone blockers is recommended according to the present evidence. Fibrinolytic therapy can be considered a reasonable option for the relatively stable ST segment elevation myocardial infarction (STEMI) patient with suspected or known COVID-19. However, primary percutaneous coronary intervention is still the standard of care in patients with definite STEMI if personal protective equipment is available and cardiac catheterization laboratory has a good infection control. In patients with elevated cardiac enzymes, it is very important to differentiate patients with Type 2 myocardial infarction or myocarditis from those with true acute coronary syndromes because invasive percutaneous intervention management in the former may be unnecessary, especially if they are hemodynamically stable. Finally, patients with baseline QT prolongation or those taking QT prolonging drugs must be cautious when treating with lopinavir/ritonavir and hydroxychloroquine for COVID-19.

## Introduction

Coronavirus disease 2019 (COVID-19) is declared a pandemic by the World Health Organization on March 11^th^ 2020. It is caused by severe acute respiratory syndrome coronavirus 2 (SARS-CoV2) 1. Its infection may directly influence cardiovascular system. Patients with preexistent cardiovascular disease may predispose to COVID-19 infection and have an elevated risk of adverse outcomes if infected 2,3. A recent meta-analysis of 8 studies from China including 46,248 patients with COVID-19 demonstrate the most prevalent comorbidities were hypertension (17 ± 7%), diabetes (8 ± 6%), and cardiovascular diseases (5 ± 4%) 4. These comorbidities are frequently noted in cardiovascular clinic. At present, the extraordinary large numbers of infected people requiring treatment may influence adequate treatment delivery to patients with acute cardiovascular disease. Pharmacological agents for COVID-19 may have some adverse cardiovascular effects. Clinicians delivering medical care including cardiovascular doctors are at risk of developing the illness or become spreader for the infection. Hence, COVID-19 infection will alter our daily practice for cardiovascular treatment greatly and have a huge impact on the routine management of cardiovascular disease. Although COVID-19 infection mainly involves the respiratory systems, cardiovascular specialists will be actively engaged in the care of patients with suspected or confirmed COVID-19. The objective of this review is to provide the latest advancements for cardiovascular disease management during the COVID-19 pandemic in different cardiovascular patients.

## Patients with cardiovascular symptoms

Although the most common symptoms of patients with COVID-19 are fever (88%) and dry cough (67.7%), there are 7.3% patients presenting with palpitation 5 and 19% patients with dyspnea 6. Additionally, patients with cardiovascular disease and COVID-19 may have overlapping symptoms. Although the mainly presenting symptoms of COVID-19 are respiratory, a case report described an Italian patient with chest pain and electrocardiographic changes for which the cardiac catheterization lab was activated. However, the patient was found to have patent coronary artery but finally tested positive for COVID-19. Palpitation, dyspnea, and chest pain, frequently noted in cardiovascular patients, may be the presenting symptoms of COVID-19 infection. Hence, when surveying the patients presented with palpitation, dyspnea, or chest pain during COVID-19 pandemic, we should especially watch out for the concomitant symptoms such as fever, chills, cough, myalgia, and fatigue which were frequently noted in COVID-19 patients 7 and contact history for differentiating patients with true cardiovascular disease from those with COVID-19 infection.

## Patients with hypertension

SARS-CoV-2 infection is caused by binding of the viral surface spike protein to the human angiotensin-converting enzyme 2 (ACE2) receptor 8, which is a membrane bound aminopeptidase that is highly expressed in the heart and lungs and has a vital role in the immune system 9. ACE2 is an enzyme responsible of the cleavage of angiotensin II into angiotensin 1-7, which has vasodilating and anti-inflammatory effects 10. ACE2 internalization by SARS-CoV-2 infection would theoretically cause the loss of ACE2 at the cell surface and decrease the degradation of angiotensin II into cardiovascular disease protective angiotensin 1-7. Indeed, an increase in the overall ratio of angiotensin II to angiotension-1-7 after ACE2 internalization may exacerbate the pulmonary tissue damage initially provoked by SARS-CoV-2 infection 11. In addition, virus-mediated down-regulation of ACE2 may enhance angiotensin II stimulation and cause the harmful hyper-inflammatory reaction of COVID-19.

If ACE2 is a functional receptor for SARS-CoV-2, the potential harmful effect and safety of ACE inhibitors or angiotensin- receptor blockers in patients with COVID-19 should be carefully considered 3. In fact, many patients from South America, Central America and Spain, have stopped or intend to interrupt their treatments with renin-angiotensin-aldosterone blockers 12. ACE2 may be up-regulated in patients treated with ACE inhibitors or angiotensin receptor blockers. ACE2 up-regulation may increase the susceptibility to COVID-19. This is particularly observed in patients with diabetes and/or hypertension, since they are usually taking ACE inhibitors or angiotensin receptor blockers 13. Currently, it is known that the effect of renin-angiotensin-aldosterone inhibitors on ACE2 is chiefly due to the expression of ACE2 in the heart, kidney and plasma, and it is not completely understood whether renin-angiotensin-aldosterone inhibitors can affect the expression of ACE2 in airway epithelial cells. To date, there is no evidence that using renin-angiotensin-aldosterone inhibitors makes patients more susceptible to the SARS-CoV-2 virus infection.

Additionally, increased renin-angiotensin-aldosterone system activity is a poor prognostic factor for severe pneumonia. Animal studies have demonstrated that renin-angiotensin-aldosterone inhibitors were able to effectively relieve symptoms of severe pneumonia. Hence, renin-angiotensin-aldosterone blockers could be useful in patients with COVID-19 pneumonia 14. Furthermore, ACE inhibitors or angiotensin receptor blockers may be protective through the inhibition of angiotensin II mediated vasoconstriction and inflammatory activation. The renin-angiotensin-aldosterone system is widely implicated in diabetes, hypertension, and heart failure. ACE inhibitors or angiotensin receptor blockers, based upon strong evidence of efficacy, are commonly used in the management of hypertension, heart failure, post myocardial infarction care, and to slow progression of renal disease. Hence, whether hypertension patients with COVID-19 who are taking renin-angiotensin-aldosterone blockers should change to another class of antihypertensive drug remains controversial. At present, there is no evidence to abandon renin-angiotensin-aldosterone blockers in patents with COVID-19 15.

## Patients needing cardiac catheterization

### Elective patients

Elective procedures may be held in order to avoid exposure of patients to the hospital where the chance of contact with COVID-19 patients may be increased. In addition, many of elective patients for cardiac catheterization may have several significant comorbidities, whose prognosis may be poorer if they are actually infected by SARS-CoV-2. However, patient decisions should be individualized, balancing the risk of COVID-19 exposure versus the risk of delay in procedure. It seems to be reasonable to avoid elective procedures on patients in whom the expected length of hospital stay is more than 3 days or intensive care unit admission is required. Furthermore, the definition of really elective procedures needs clinical judgement, because in some patients delayed elective procedures may have harmful effects. Examples of elective procedures delayed reasonably include coronary angiography examination or percutaneous coronary intervention (PCI) for stable ischemic heart disease, pre-operation survey for stable valvular heart disease, endovascular intervention for stable peripheral artery occlusion disease, transcatheter aortic valve implantation for stable aortic stenosis, and so on.

### Patients with ST-segment elevation myocardial infarction (STEMI)

Because rapid nucleic acid testing for SARS-CoV-2 infection is not available in many hospital, the treatment strategy of STEMI patients with suspected or known COVID-19 is needed to be modified 16,17. Although primary PCI is the routine for STEMI patients in most hospital with cardiac catheterization laboratory, fibrinolytic therapy can be considered a reasonable option for the relatively stable STEMI patient with suspected or known COVID-19 16,17 in order to decrease staff exposure to possible SARS-CoV-2 infection. Very few cardiac catheterization laboratories are equipped with negative ventilation systems and, thus, the risk of transmission remains high with each encounter 18. Based on such safety concerns and along with the acceptable mortality benefit of the new generation of fibrinolytic agents, Sadeghipour et al. reported that thrombolytic therapy as a potential first choice on several occasions during the outbreak is acceptable 19.

**Figure 1:**
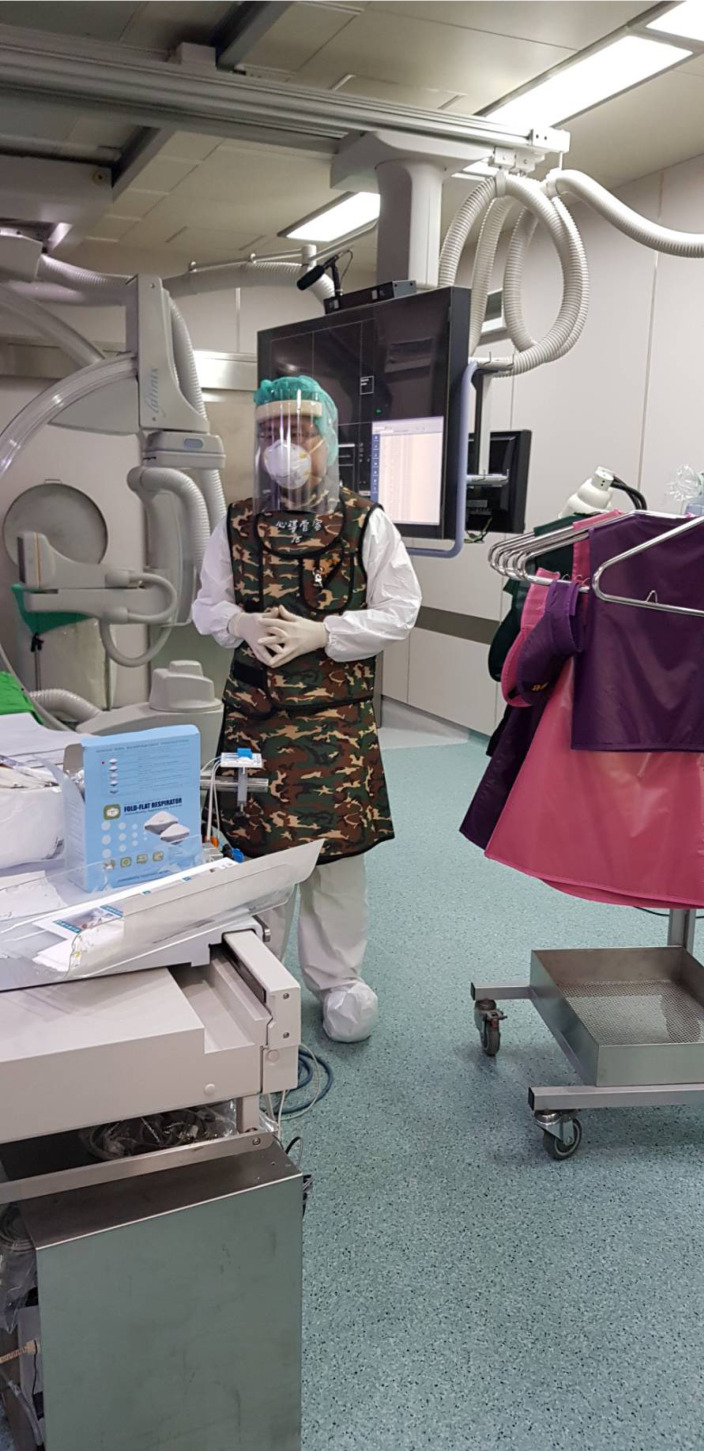
During the procedure of percutaneous coronary intervention for patients with suspected or known COVID-19 infection, appropriate personal protective equipment including gloves, gown, shield, and a N95 mask must be worn.

In contrast, Mahmud et al. raised the opposed opinion recently 20. They emphasize that primary PCI should still remain the standard of care of patients with definite STEMI, including for COVID-19 confirmed or probable patients. Compared to thrombolytic therapy, primary PCI is superior for restoring normal coronary flow and has a significantly lower risk of fatal and nonfatal bleeding complications 21. In addition, after a fibrinolysis-based strategy, just around 50% of patients reperfuse adequately and, thus, a high proportion of patients require rescue PCI 21. This can cause prolonged intensive care unit stay and limiting access of intensive care unit beds for COVID-19 patients. Hence, every primary PCI center will need to monitor the ability to provide timely primary PCI based on staff and personal protective equipment availability. During the procedure of primary PCI, appropriate personal protective equipment must be worn including gloves, gown, shield, and a N95 mask (**Figure 1**). Furthermore, most of catheterization laboratories are not designed for infection isolation and only have positive ventilation system, so catheterization laboratories will need a complete clean after the procedure. In the absence of these resources, a fibrinolysis first approach should be considered.

### Patients with non-ST segment elevation myocardial infarction (NSTEMI) or unstable angina

For most patients with NSTEMI or unstable angina and suspected COVID-19, SARS-CoV-2 infection should be confirmed first. The confirmed patients must be transferred to the isolation ward. After test of SARS-CoV-2 infection becomes negative, it is assessed whether further invasive interventions are needed. Very few patients with NSTEMI or unstable angina and suspected COVID-19 may have unstable hemodynamic and fatal arrhythmia and need urgent PCI. Similarly, appropriate personal protective equipment must be worn during this procedure 17. In addition, in hemodynamically stable patients with NSTEMI or unstable angina and excluded COVID-19, quick discharge after revascularization is likely important in terms of decreasing patient exposure within the hospital.

## Patients with cardiac enzyme elevation

Acute cardiac injury is present in 7-13 % of patients with COVID-19 and may belong to either type 2 myocardial infarction (MI) or myocarditis 1,22,23. It is very important to differentiate patients with Type 2 MI or myocarditis from those with true acute coronary syndromes because invasive PCI management in the former may be unnecessary, especially if they are hemodynamically stable.

## Patients with arrhythmia

In hospitalized COVID-19 patients, cardiac arrhythmia was found in 16.7% of 138 patients 22. Unfortunately, most of arrhythmia might be caused by metabolic disarray, hypoxia, neurohormonal, or inflammatory stress in the setting of viral infection. Nevertheless, patients with palpitation or eletrocardiography showing arrhythmia may be the presenting symptom/sign of COVID-19 infection.

## Patients with heart failure

Zhou et al. found heart failure was noted in 23.0% patients with COVID-19 24. Furthermore, heart failure was more common in mortality patients than in survival patients of COVID-19 infection. Worsening heart failure may be due to exacerbation of pre-existing left ventricular dysfunction or new myocarditis remains unclear. Patients with COVID-19 may have right side heart failure and associated pulmonary hypertension, especially in those with severe pneumonia. Hence, COVID-19 infection should be suspected in heart failure patients with acute exacerbation.

## Patients with QT prolongation

Although there are no definitely effective therapies for COVID-19, various pharmacologic agents are under active study. Several antiviral agents are the first medications under study for the treatment of COVID-19. Among these agents, lopinavir/ritonavir may lead to QT prolongation, especially in patients who have a baseline long QT interval or those taking other QT prolonging drugs 25. In addition to anti-viral therapy, in a small sample size study, hydroxychloroquine treatment is significantly associated with viral load reduction/disappearance in COVID-19 patients and its effect is reinforced by azithromycin 26. These two drugs may also prolong QT interval 27,28. Hence, patients with baseline QT prolongation or those taking QT prolonging drugs must be cautious when treating with the above medicine for COVID-19.

## Conclusions

Based on clinical presentation, pathophysiology, high infectivity, high cardiovascular involvement, and therapeutic agents with cardiovascular toxicity of COVID-19, regular cardiovascular treatment is being changing greatly. Despite ACE2 serving as the portal for infection, the continuation of clinically indicated renin-angiotensin-aldosterone blockers is recommended according to the present evidence. Fibrinolytic therapy can be considered a reasonable option for the relatively stable STEMI patient with suspected or known COVID-19. However, primary PCI is still the standard of care in patients with definite STEMI if personal protective equipment is available and cardiac catheterization laboratory has a good infection control. In patients with elevated cardiac enzymes, it is very important to differentiate patients with Type 2 MI or myocarditis from those with true acute coronary syndromes because invasive PCI management in the former may be unnecessary, especially if they are hemodynamically stable. Finally, patients with baseline QT prolongation or those taking QT prolonging drugs must be cautious when treating with lopinavir/ritonavir and hydroxychloroquine for COVID-19.
